# β-catenin-independent WNT signaling and Ki67 in contrast to the estrogen receptor status are prognostic and associated with poor prognosis in breast cancer liver metastases

**DOI:** 10.1007/s10585-016-9780-3

**Published:** 2016-02-09

**Authors:** Annalen Bleckmann, Lena-Christin Conradi, Kerstin Menck, Nadine Annette Schmick, Antonia Schubert, Eva Rietkötter, Jetcy Arackal, Peter Middel, Alexandra Schambony, Torsten Liersch, Kia Homayounfar, Tim Beißbarth, Florian Klemm, Claudia Binder, Tobias Pukrop

**Affiliations:** Department of Hematology/Medical Oncology, University Medical Center Göttingen, 37099 Göttingen, Germany; Department of Medical Statistics, University Medical Center Göttingen, 37099 Göttingen, Germany; Department of General, Visceral and Pediatric Surgery, University Medical Center Göttingen, 37099 Göttingen, Germany; Institute of Pathology, University Medical Center Göttingen, 37099 Göttingen, Germany; Department Biology, Developmental Biology, Friedrich-Alexander University Erlangen-Nürnberg, 91058 Erlangen, Germany; Clinic for Internal Medicine III, Hematology and Medical Oncology, University Regensburg, 93053 Regensburg, Germany

**Keywords:** Breast cancer, Metastasis, WNT signaling, Prognostic score

## Abstract

Liver metastasis development in breast cancer patients is common and confers a poor prognosis. So far, the prognostic significance of surgical resection and clinical relevance of biomarker analysis in metastatic tissue have barely been investigated. We previously demonstrated an impact of WNT signaling in breast cancer brain metastasis. This study aimed to investigate the value of established prognostic markers and WNT signaling components in liver metastases. Overall N = 34 breast cancer liver metastases (with matched primaries in 19/34 cases) were included in this retrospective study. Primaries and metastatic samples were analyzed for their expression of the estrogen (ER) and progesterone receptor, HER-2, Ki67, and various WNT signaling-components by immunohistochemistry. Furthermore, β-catenin-dependent and -independent WNT scores were generated and analyzed for their prognostic value. Additionally, the influence of the alternative WNT receptor ROR on signaling and invasiveness was analyzed in vitro. ER positivity (HR 0.09, 95 % CI 0.01–0.56) and high Ki67 (HR 3.68, 95 % CI 1.12–12.06) in the primaries had prognostic impact. However, only Ki67 remained prognostic in the metastatic tissue (HR 2.46, 95 % CI 1.11–5.44). Additionally, the β-catenin-independent WNT score correlated with reduced overall survival only in the metastasized situation (HR 2.19, 95 % CI 1.02–4.69, *p* = 0.0391). This is in line with the in vitro results of the alternative WNT receptors ROR1 and ROR2, which foster invasion. In breast cancer, the value of prognostic markers established in primary tumors cannot directly be translated to metastases. Our results revealed β-catenin-independent WNT signaling to be associated with poor prognosis in patients with breast cancer liver metastasis.

**Electronic supplementary material:**

The online version of this article (doi:10.1007/s10585-016-9780-3) contains supplementary material, which is available to authorized users.

## Introduction

The 5-year overall survival (OS) for breast cancer patients in Europe increased over time from 78.4 % (1999–2001) to 82.4 % (2005–2007) owing to the availability of more efficient treatment modalities nowadays [1]. However, the overall survival decreases dramatically to only 2–3 years for patients on diagnosis of distant metastasis [2]. Besides bone, the liver is the most frequent site of breast cancer metastasis with an incidence of 40–50 % of all metastasized patients [3]. Nevertheless, resection of isolated breast cancer liver metastases is still a controversial topic of discussion. However, this locoregional treatment is a well-established approach in a multimodal therapeutic concept for patients with metastasized colorectal cancer [3–8]. Probable reasons for this controversial debate are the diversity of the metastatic pattern in different organs, the lack of prognostic biomarkers in this situation, and the heterogeneity of breast cancer. The pattern of organ metastasis is partially determined by features of the primary tumor cells [9, 10]. For example, hormone-receptor-positive breast carcinoma cells rather metastasize into bone tissue, while triple-negative breast cancer cells initially spread to other solid organs, such as the liver for example. A number of molecular characteristics in the carcinoma cells have already been identified for this organo-tropism [9, 11]. Furthermore, the last steps of metastasis in host organs, such as colonization and macroscopic outgrowth, are influenced by the unique environments of the target organs of metastasis [12].

Recent genomic analyses of primary tumors in comparison to metastatic tissue indicated that the first steps of metastasis including seeding into distant organs are early events and thus the metastatic cells appear to go through an evolutionary process in parallel to the primary tumor (*parallel progression or branched evolution*) [13–16]. Fortunately, many seeded carcinoma cells undergo apoptosis in the microenvironment of the foreign host organ and only few carcinoma cells colonize successfully [17–20]. Thus, the final steps of metastasis are the most vulnerable and least effective during this process and are massively influenced by its own genetic evolution and the specific metastatic microenvironment.

We recently demonstrated in breast cancer with brain metastasis that the local defense system of the brain, composed of astrocytes and microglia, attempts to combat the epithelial carcinoma cells foreign to the brain. This glial attack leads to apoptosis in some cancer cells. However, during this defense program the carcinoma cells also benefit from molecules secreted by the microenvironment that enhance their invasion. Further analysis revealed that WNT signaling is involved in this glia-induced carcinoma cell invasion during colonization of the brain tissue, indicating some brain-specific activation of WNT [18, 21]. In addition, immunohistochemistry of brain metastases of non-small cell lung cancer (NSCLC) [22] and breast cancer [23] confirmed the role of WNT signaling in macroscopically established brain metastases. However, both evaluations indicated an important role for components of the β-catenin-independent WNT pathway. Comparable paracrine WNT activation of colonizing carcinoma cells by the metastatic microenvironments at other sites is likely; however this has not been analyzed systematically.

The involvement of WNT signaling in the process of metastasis is not unexpected, since the WNT pathways regulate important events such as tumor initiation, carcinoma cell migration/invasion, epithelial mesenchymal transition (EMT), angiogenesis, lymphangiogenesis, and wound healing. In addition, WNT components are significantly involved in many embryonic development processes [24–26]. Interestingly, in individual developmental steps, for example during heart development, it is necessary that phases of active WNT signals are interchanged by phases of WNT inhibition in a specific time sequence [27]. Certain parallels may be assumed for metastasis. In the early metastatic steps, the cancer cells perform an EMT-like process known to be governed by active WNT/β-catenin signaling. Briefly, EMT includes, among others, the down-regulation of E-cadherin, the activation and translocation of β-catenin into the nucleus. There it regulates the WNT/β-catenin target genes as a co-transcription factor together with the transcription factors of the lymphoid enhancer factor/T cell factor (LEF/TCF) family. However, in the distant organ, the reverse process of EMT—mesenchymal epithelial transition (MET)—is presumed to take place. A recent study demonstrated the repression of the EMT inducer Prrx1 as a mandatory prerequisite for successful metastatic colonization of the lung [28] and that up-regulation of E-cadherin could accelerate this colonization by improving the intercellular exchange of growth factors between the metastatic carcinoma cells [29]. Moreover, in contrast to colon cancer cell lines in breast cancer cells the WNT/β-catenin activity is low to not measurable [30–32].

For these reasons, it is not unexpected that alternative WNT signals suppressing WNT/β-catenin-signaling are detected in breast cancer as well as during the final steps of metastasis in distant organs. Our recent studies of human brain parenchyma colonization and metastasis revealed overexpression of WNT5a/b, ROR1/ROR2, increased activity of WNT/c-Jun and not active β-catenin [23]. ROR1/ROR2 belong to the receptor tyrosine kinases (RTK) and are activated by the binding of WNT5a [33, 34]. The activation of the kinase domain leads to Jun-N-terminal kinase (JNK) and subsequent c-Jun activation. Interestingly, WNT5a/ROR1/ROR2-dependent signaling can also lead to WNT/β-catenin inhibition. Additionally, the ligand WNT5a can also act through Frizzled (Fz) receptors and the phosphoprotein dishevelled (DVL), ultimately activating the so-called WNT/planar cell polarity pathway (WNT/PCP) [35, 36]. WNT/PCP signaling is very important in the organization of tissue polarity, ensuring the correct orientation of a single epithelial cell within the organization and function of the whole tissue. Thus it is not surprising that components of the WNT/PCP pathway are aberrantly overexpressed during the establishment of malignant epithelial tissue in hitherto unforeseen organs.

Taken together, it may be assumed that the biological features of the carcinoma cells, including their WNT activity, have to change during the various steps of metastasis to allow successful adjustment to the current conditions/microenvironment, otherwise the carcinoma cells will undergo apoptosis. In accordance with these assumptions, clinical and pathological scores, determined in the tissue of the primary tumor, cannot simply be transferred to the metastatic tissue. However, pathological scoring systems relate almost solely on studies of the primary tumor tissues such as the most prominent predictive markers in breast cancer, the estrogen receptor (ER), the progesterone receptor (PGR) and the erb-b2 receptor tyrosine kinase (HER-2) status. Furthermore, the triple-negative subtype has a negative prognostic impact. Additionally, the proliferation status quantified by Ki67 is also of prognostic value. Again, these markers are mostly determined in the primary tumor and their prognostic capacity determined in metastatic tissue remains poorly defined. This can be attributed to the fact that the clinical routine in breast cancer patients does not include metastatic surgery or biopsy of the metastatic tissue and thus the availability of matched tissue samples of the primary tumor and metastatic tissue derived thereof is rare.

Thus, the aim of this study was to investigate the value of established prognostic markers (such as ER, PGR, HER-2) and WNT components in liver metastases of breast cancer and matched primaries. This work is based on in vitro data analyzing the effects on signaling and invasion of β-catenin-independent WNT signaling via the alternative WNT receptor ROR.

## Materials and methods

### Cell lines and cell culture

If not indicated otherwise, all reagents and chemicals were purchased from Sigma (Munich, Germany). The human breast cancer cell lines MCF-7, MDA-MB-231 and SK-BR-3 were obtained from the American Type Culture Collection (ATCC, Rockville, USA) and were cultured in RPMI-1640 media (PAA, Cölbe, Germany) supplemented with 10 % fetal bovine serum (FCS; Sigma, Munich, Germany).

### Knockdown and overexpression

To generate ROR1 shRNA lentiviral particles, HEK293T cells (ATCC) were co-transfected with the packaging plasmids pMD2.G (Addgene plasmid: 12259) and pCMVΔR8.2 (Addgene plasmid: 12263, both provided by Didier Trono, École Polytechnique Fédérale de Lausanne, Laboratory of Virology and Genetics, Lausanne, Switzerland) and either the pGIPZ non-silencing control (ns ctl) shRNA or shROR1 plasmid (Thermo Scientific, Schwerte, Germany) through calcium phosphate precipitation. While the ns ctl sequence is proprietary, the mature ROR1 targeting sequence is 5′-ATTTATAGGATCTGCCATG-3′. Virus-containing supernatants were concentrated using lentiviral enrichment reagent (MobiTech, Göttingen, Germany) and viral titers were calculated based upon the GFP expression of HEK293T transduced with serial dilutions of the shRNA of interest. MDA-MB-231 cells were finally transduced with a multiplicity of infection of 5.0. Cells were selected in medium with 2 μg/mL puromycin (Sigma, Munich, Germany).

For ROR2 overexpression, the plasmids pcDNA 3.1/Zeo(+) (Invitrogen, Paisley, UK) and pcDNA-hsROR2 were introduced into MCF-7 and SK-BR-3 cells using the Nanofectin transfection reagent (PAA, Cölbe, Germany). Stable expression was achieved by selecting for zeomycin (100 µg/ml) resistance.

### RNA isolation and qRT-PCR

RNA was isolated using the High Pure RNA isolation kit (Roche, Mannheim, Germany). Reverse transcription was accomplished with the iScript Master Mix (BioRad, Munich, Germany). QRT-PCR was performed using SYBR green detection with mRNA-specific primers (Supplemental Table 1) on the ABI PRISM 7900HT system (Applied Biosystems, Darmstadt, Germany). Gene expression was analyzed with SDS, software version 2.4 (Applied Sciences) and normalized to the two housekeeping genes *GNB2L1* and *HPRT1*.

### Sub-cellular fractionation, protein lysis, immunoprecipitation, and immunoblot

To analyze the sub-cellular localization of our proteins of interest, cytosolic and nuclear fractions of cells were isolated as follows: after washing in PBS, cells were resuspended in cold hypotonic buffer (10 mM HEPES, pH 7.9, 1.5 mM MgCl_2_, 10 mM KCl, 0.5 mM DTT) and lysed by the addition of 0.5 % (v/v) Nonidet P-40, vortexed and centrifuged at 750 g for 1 min at 4 °C. The resulting supernatant was then collected as the cytosolic extract. The remaining pellet was resuspended in extraction buffer (20 mM HEPES, pH 7.9, 420 mM NaCl, 15 mM MgCl_2_, 0.2 mM EDTA, 25 % (v/v) glycerol, 1 % (v/v) Nonidet P-40, 0.5 % (v/v) sodium deoxycholic acid, 0.5 mM DTT), incubated for 30 min at 4 °C, and centrifuged at 5000 g for 5 min at 4 °C. The supernatant was collected as the nuclear extract. In order to confirm a successful fractionation, all fractions were routinely tested for the expression of HDAC which should only be present in the nuclear fraction. For whole cell lysate preparation, cells were treated with RIPA lysis buffer (50 mM Tris, pH 7.2, 150 mM NaCl, 0.1 % (v/v) SDS, 0.5 % sodium deoxycholic acid, 1 % (v/v) Triton X-100). All buffers were supplemented with protease inhibitors (Sigma) as well as phosphatase inhibitors (Roche). Protein quantification was carried out with the D_C_ protein assay (Bio-Rad, Munich, Germany). For co-immunoprecipitations, MCF-7 cells were transiently transfected with plasmids encoding ROR2-Flag [37] and/or Dvl1-myc, Dvl2-myc or Dvl3-myc [38] using Lipofectamine LTX (Thermo Fisher Scientific, Braunschweig, Germany) according to the manufacturer’s instructions. Twenty-four hours post transfection, cells were stimulated for 45 min with either control supernatant or supernatant of Wnt5a-overexpressing cells. Wnt-5a conditioned medium was collected from 3T3 murine fibroblasts infected with pMSCV-Xenopus Wnt-5a or an empty control vector. Cells were lyzed in 10 mM HEPES pH 7.4, 150 mM NaCl, 0.5 % NP-40 and 0.5 % OGP supplemented with protease and phosphatase inhibitors. Up to 750 µg protein were used for immunoprecipitation with an anti-Flag antibody (#8146, cell signaling) and anti-Flag M2 magnetic beads (Sigma) according to standard protocols.

The obtained lysates were subjected to immunoblotting and proteins were detected with antibodies specific to WNT5a (#MAB645, R&D Systems, Wiesbaden, Germany), ROR2, total β-catenin (#sc-98486,#sc-7963, Santa Cruz, Heidelberg, Germany), active β-catenin, Tubulin (#05-665,#05-829, Merck Millipore, Darmstadt, Germany), ROR1, myc, Flag, Dvl3, c-Jun or HDAC1 (#4102,#2276,#8146,#3218,#9165,#2062, Cell Signaling, Frankfurt, Germany). All immunoblots were carried out in three technically and biologically independent experiments.

### Flow cytometry

Cell lines were stained with a PE-conjugated monoclonal antibody against human ROR1 (#357803, BioLegend) according to the manufacturer’s instructions. An irrelevant IgG1 antibody was used as respective isotype-matched negative control (BioLegend). Fluorescence was measured with a FACSCanto II flow cytometer (BD Biosciences, Heidelberg, Germany). Flow cytometry results were analyzed using Kaluza, software version 1.2 (Beckman Coulter, Krefeld, Germany).

### In-vitro invasion and proliferation assays

The invasive capacity of the cells was measured in a modified Boyden chamber as previously published [39]. Briefly, 1 × 10^5^ cells were seeded in triplicate on an ECM-coated (R&D systems, Wiesbaden, Germany) polycarbonate membrane (pore diameter 10 µm, Nucleopore, Tübingen, Germany) and grown for 96 h [39]. Cell invasion was quantified by counting the number of invasive cells in the lower wells and relating it to the wildtype control. Viability and real-time proliferation were analyzed using the xCELLigence RTCA DP system (Roche, Mannheim, Germany). For this purpose, 4 × 10^4^ cells were seeded per well in quadruplets and analyzed for 96 h. All invasion and proliferation assays were carried out in three biologically independent experiments.

### Immunofluorescence staining

Cells were stained as previously described using the above mentioned antibodies and were analyzed with either a confocal laser scanning microscope (LSM 510, Zeiss, Göttingen, Germany) (Pukrop et al. [21]) or a conventional fluorescence microscope (DM5000B, Leica Microsystems, Wetzlar, Germany).

### Human tissue samples of hepatic metastases and primary tumors

Formalin-fixed paraffin-embedded (FFPE) hepatic metastases and primary breast cancer samples from patients treated at the University Medical Center Göttingen between 1998 and 2011 were obtained from the local Pathology Department. In total, 34 hepatic metastases (either from punch biopsies (n = 27) or resection specimens after liver resection (n = 6)) and 19 matched primary tumors (surgical resection specimens) were available for immunohistochemical analyses (Supplemental Fig. 1B). All patient samples were collected following approval by the local ethics committee (vote: 21/3/11).

### Study cohort

The patient cohort was characterized in terms of demographics, clinical baseline data, and treatment regimens.

Follow-up examinations were performed according to individual physicians’ discretion and data were obtained either from the local clinical cancer registry or the treating physician. OS after primary surgical treatment (OS primary tumor) was defined as the interval between the surgical resection of the primary tumor and cancer-related death. Survival after liver metastasis (OS liver metastasis) was defined as the interval between the surgical resection or biopsy of liver metastasis and death, which was cancer-related in all cases.

### Immunohistochemistry (IHC)

Immunohistochemical analyses were performed using formalin-fixed, paraffin-embedded (FFPE) tissue samples cut into 2-µm-thick slices and stained on a Ventana BenchMark XT immunostainer (Ventana, Tucson, AZ, US) according to standardized protocols.

Estrogen (ER), progesterone (PGR), HER-2 and proliferation index Ki67 were determined (ER and PR were available for all specimens from the routine histopathological work-up) using immunohistochemical staining. For HER-2 staining, a standardized immunohistochemical staining technique was performed using a PATHWAY^®^ anti-HER-2 (4B5) rabbit monoclonal antibody (Ventana Medical Systems, Mannheim, Germany). Heat epitope retrieval using the immunostainer was performed for 60 min at 100 °C. The anti-HER-2 antibody was incubated at 37 °C for 32 min. Enzymatic reactivity was visualized by means of horseradish peroxidase with the ultraView Universal DAB Detection Kit (Ventana Medical Systems). HER-2 gene amplification was detected using the Ventana INFORM HER-2 Dual ISH/DNA Probe Cocktail and visualized utilizing two-color chromogenic in situ hybridization (ultraVIEW SISH Detection KIT and ultraVIEW Red ISH DIG Detection Kit, Ventana Medical Systems). The Ki67 antibody used is also a monoclonal mouse antibody (Zytomed Systems, code number MSK018) and was diluted to 1:500.

Purified mouse anti-E-cadherin (BD Biosciences, Heidelberg, Germany) was used after treatment with CC1 for 60 min at a dilution of 1:400 for 30 min. The mouse antibody β-catenin (E-5) sc-7963 (Santa Cruz Biotechnology Inc., Heidelberg, Germany) was used at a dilution of 1:200 for 30 min to detect β-catenin. Phospho-c-Jun was visualized by means of the polyclonal Phospho-c-Jun (Ser63) II antibody (Cell Signaling Technology^®^, Massachusetts, USA) at a dilution of 1:50 for 80 min. Heat epitope retrieval was done with CC2 treatment for 64 min. The OptiView DAB IHC Detection Kit (Ventana Medical Systems, Tucson, Arizona, USA) was used as a secondary antibody for E-cadherin, β-catenin and Phospho-c-Jun. For the immunohistochemical staining of c-Jun, the monoclonal antibody c-Jun (60A8) Rabbit mAB#9165 (Cell Signaling Technology^®^, Massachusetts, USA) was used after demasking with CC1 for 60 min. LEF-1 was demasked with CC1 for 90 min and stained for 90 min with the monoclonal antibody Lef1 (C18A7) Rabbit mAb#2286 (Cell Signaling Technology^®^, Massachusetts, USA) at a dilution of 1:50. The polyclonal antibody Dvl3#3218 (Cell Signaling Technology^®^, Massachusetts, USA) was used at a dilution of 1:50 for 90 min after preconditioning with CC1 for 90 min for the determination of Dvl3.

### Analyses of immunohistochemical stainings and definition of WNT scores

Tumor cell staining alone was evaluated; microenvironment staining was ignored. For the hormone receptor expression membrane staining activity for ER and PR was determined and rated as positive when ≥10 % of tumor cells were positive. Furthermore, the accurate nuclear staining percentage was assessed for Ki67. Owing to the fact that almost all primary and metastases samples were positive for the proliferation index Ki67, the median expression was taken as cutoff to perform survival analyses.

HER-2 expression was scored according to established histopathological guidelines for breast cancer. In the case of equivocal staining, additional slides were prepared for chromogene or silver in situ hybridization (C/S-ISH) [40]. In-situ hybridization was used to reveal gene amplification in specimens scored as IHC2+ and to confirm gene amplification in all IHC3+ cases. Ratios of >2.2 indicated HER-2 gene amplification. In the case of an equivocal result for gene amplification (ratio 1.8–2.2), additional cells (at least 20 additional cancer cells) were analyzed. The HER-2 status was defined as positive if tissue samples were scored as IHC2+/SISH+ or IHC3+ and negative if they were scored as IHC0, IHC1+ or IHC2+/S-ISH−.

For all other stainings forming the WNT scores, antigene immunoreactivity was scored for nuclear staining (Dvl3, Lef1, β-catenin, Phospho-c-Jun, c-Jun); membrane staining was analyzed for E-cadherin. Staining intensity was assessed and the proportion of positive cells was documented when at least 300 tumor cells were accessible. The slides were screened at a low magnification for the pattern and distribution of the staining. The tumor cell percentage was categorized into five groups: 0 = 0 %; 1+ = (1–25 %); 2+ = (26–50 %); 3+ = (51–75 %); 4+ = (76–100 %). For the WNT Score nuclear (Dvl3, Lef1, β-catenin, Phospho-c-Jun, c-Jun) and membrane (E-cadherin) positivity was used based on the percentage of positive tumor cells. Both WNT scores are the sum of the IHC expression of different WNT components and were calculated as follows: β-catenin-dependent WNT score = staining score_nuclear β-catenin_ + staining score_nuclear Lef1_ + inverse staining score_E-cadherin_. Due to the fact, that membrane E-cadherin levels are down-regulated during EMT/active β-catenin-dependent WNT we used for this WNT score the E-cadherin inverse staining score (membrane). We state, that during active β-catenin-dependent WNT signaling membrane E-cadherin levels change (e.g. decrease), but the signaling is not independent of E-cadherin.

The β-catenin-independent WNT score we calculated as follows: β-catenin-independent WNT score = staining score_nuclear Dvl3_ + staining score_nuclear c-Jun_ + staining score_nuclear Phopho-c-Jun_. All slides were evaluated independently by two different observers, who remained blind to the patient data and clinical outcome. The standardized manner of specimen preparation was performed according to the REMARK guidelines for biomarker studies [41].

### Bioinformatics methods and statistical data analysis

Survival analysis was performed for OS following surgery of the primary tumor (OS primary tumor) as well as following histologically confirmed diagnosis of liver metastasis (OS liver metastasis). Events were defined as cancer-related death; all other events were considered as censored. Survival data were visualized using Kaplan–Meier plots and significance was calculated using the logrank test for univariate analyses. *p* values < 0.05 were considered significant. Pearson‘s correlation test was used to calculate correlation of expression changes in the breast cancer primaries and matched liver metastases.

All analyses were performed using the free statistical software R (version 2.15.1; http://www.r-project.org).

## Results

### ROR1 is overexpressed in basal-like MDA-MB-231 cells

**Fig. 1:**
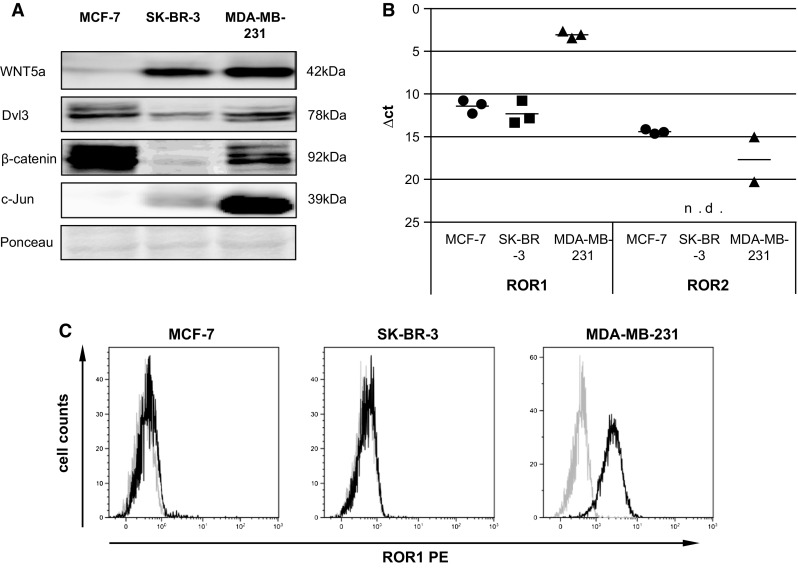
**a** Immunoblot for WNT5a, Dvl3, β-catenin, and c-Jun of MCF-7, SK-BR-3 and MDA-MB-231 cell lysates. **B** qRT-PCR of MCF-7, SK-BR-3 and MDA-MB-231 for ROR1 and ROR2. Each *dot* represents one independent biological sample (means, n = 3, n.d. = not detectable). Note that in MDA-MB-231 ROR2 was only detectable in 2/3 samples. **C** Flow cytometry (*gray* isotype control, *black* stained cells for MCF-7, SK-BR-3 and MDA-MB-231) measuring the expression of ROR1

First we characterized the WNT repertoire of the three model breast cancer cell lines MCF-7, SK-BR-3, and MDA-MB-231 representing the luminal, ERBB2/HER-2+, and basal-like molecular subtypes of breast cancer. Dvl3, which is known to be essential for WNT signaling in general, was expressed in all three cell lines. While the MDA-MB-231 revealed only moderate expression of β-catenin, the expression of WNT5a as well as c-Jun was more prominent in the MDA-MB-231 indicating β-catenin-independent WNT signaling (Fig. 1a, Supplemental Fig. 2A). Moreover, qRT-PCR revealed the β-catenin-independent WNT receptor ROR1 to be overexpressed in MDA-MB-231, whereas ROR2 was only very weakly expressed in the cell lines and even undetectable in SK-BR-3 cells (Fig. 1b). This overexpression of ROR1 in the MDA-MB-231 was confirmed by flow cytometry (Fig. 1c). Considering that the most aggressive, basal-like cell line MDA-MB-231 expressed the highest amounts of β-catenin-independent WNT proteins while active β-catenin was found at high levels in the benign, weakly invasive MCF-7 cells as well, these findings further hint towards the importance of the non-canonical signaling cascade for tumor progression.

### ROR2 overexpression and ROR1 knockdown

**Fig. 2:**
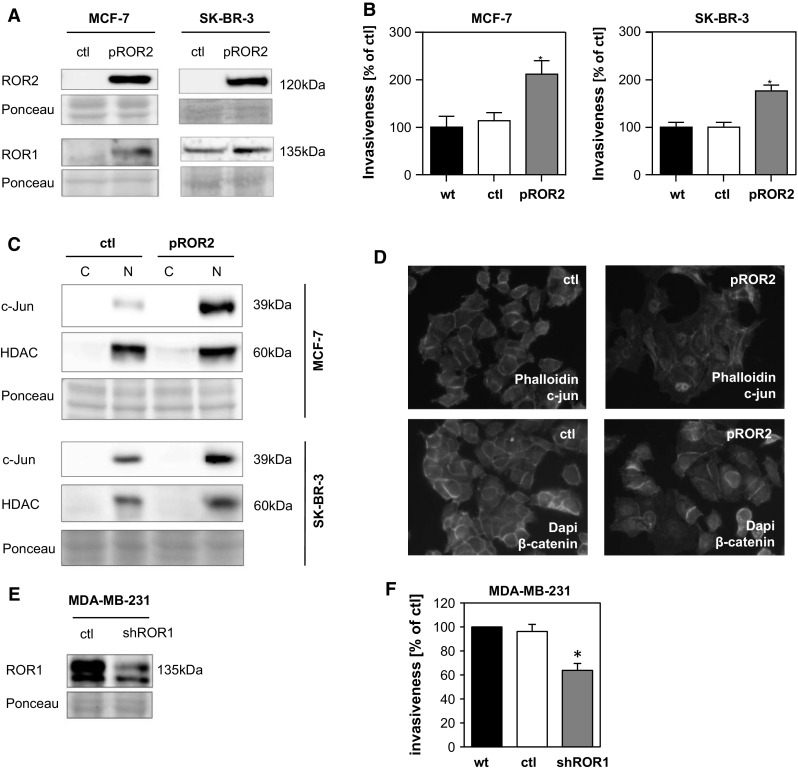
**a** Immunoblots showing the expression of ROR1 and ROR2 in MCF-7 and SK-BR-3 cells transfected with either an empty vector (ctl) or a ROR2 overexpression plasmid (pROR2). **B** In vitro microinvasion assays of ROR2-overexpressing MCF-7 and SK-BR-3 cells compared to wildtype cells (wt) or cells transfected with an empty vector (ctl) (mean ± SD, n = 3, *p < 0.001). **C** Immunoblot for c-Jun and HDAC of MCF-7 and SK-BR-3 empty vector (ctl) or ROR2-overexpressing cells for cytoplasmic (C) and nuclear (N) cell lysates. **D** Immunofluorescence staining of c-Jun and F-actin (phalloidin) in MCF-7 empty vector (ctl) and ROR2 cells as well as β-catenin and Dapi in MCF-7 wt and ROR2 cells. **E** Immunoblot of ROR1 in MDA-MB-231 cells transfected with nonsense control (ctl) or ROR1 (shROR1) shRNA. **F** In vitro microinvasion assay of MDA-MB-231 wildtype (wt), non-sense control (ctl) and ROR1 knockdown (shROR1) cells (mean ± SD, n = 3, *p < 0.001)

Previously, we demonstrated overexpression of the homologues receptors ROR1 and ROR2 in brain metastasis of breast cancer patients [24]. To further investigate the impact of ROR1/2 overexpression in breast cancer cells, we transfected both the luminal A breast cancer cell line MCF-7 and the ERBB2/HER-2+ cell line SK-BR-3 with a ROR2 construct. Transfection efficiency was verified by immunoblots (Fig. 2a) and ROR2 localization was determined through immunofluorescence staining (Supplemental Fig. 2B). Interestingly, ROR2 overexpression also resulted in an increased expression of ROR1 (Fig. 2a). We then tested the biological behavior of the cell lines. Invasive capacity was greatly increased in the ROR2 overexpressing cells compared to the empty vector control (Fig. 2b) without any changes in cell proliferation (Supplemental Fig. 2C). In the subsequent step, the ROR2 overexpressing MCF-7 and SK-BR-3 cells were characterized with regard to WNT downstream targets using immunoblotting (Fig. 2c). In comparison to the control cells, c-Jun was enriched in the ROR2 overexpressing cells, confirming an activation of β-catenin-independent WNT signaling. This can be verified by immunofluorescence, which confirms an increase in nuclear c-Jun levels, whereas the β-catenin staining depicts a cytosolic and membrane localization (Fig. 2d). In contrast, neither an influence on the levels nor the activation of β-catenin could be detected (Supplemental Fig. 2d). As a proof of concept that β-catenin-independent WNT signaling via ROR1/2 indeed mediates invasiveness, a ROR1 knockdown was performed in the marked ROR1-expressing cell line MDA-MB-231 and effectiveness validated by immunoblot and flow cytometry (Fig. 2e, Supplemental Fig. 2E). ROR2 protein expression remained undetectable in MDA-MB-231 (Supplemental Fig. 2F). Knockdown of ROR1 significantly decreased the invasive capacity of the cells compared to the control (Fig. 2f), thereby confirming a pro-invasive role for ROR1.

Since it is known that ROR1/2 signaling, especially after stimulation with Wnt5a, requires Dvl for signal transduction [42, 43], we aimed to analyze which of the Dvl proteins (Dvl1, Dvl2 or Dvl3) might be a good marker for active ROR signaling in breast cancer. Therefore, we transiently co-transfected MCF-7 cells with Flag-tagged ROR2 and myc-tagged Dvl1, Dvl2 or Dvl3 and performed co-immunoprecipitation after stimulation with Wnt5a. Interestingly, all Dvl proteins were found to interact with ROR2 independent of stimulation with Wnt5a (Supplemental Fig. 2G). Considering that among the Dvl proteins, Dvl3 has already been shown to be highly expressed in cancer patient samples [44–46] and to affect the biological behavior of lung cancer cells mainly through p38 and JNK pathway [47], we decided to include Dvl3, together with c-Jun, as markers for β-catenin independent WNT signaling.

### Characterization of the patient cohort

The patient cohort was characterized in terms of demographics, clinical baseline data, and treatment concepts according to the parameters listed in Tables 1 and 2. In total, 34 hepatic metastases and 19 matched primary tumors were characterized and further classified by IHC (see Materials and methods and Supplemental Fig. 1b).

**Table 1:** Univariate analysis of clinicopathological baseline data and IHC markers in the breast cancer primaries and their effect on overall survival (OS of the primary breast cancer since first diagnosis)

Parameter	Classification	Distribution	Impact on survival Hazard ratio [95 %-CI]	*p* value (logrank)
Age	Median [95 % CI]	57.5 [95 % CI 41.9–76.6]	Age > 57.6: 2.1695 % CI [1.02–4.57]	**0.04045**
Other distant metastases present at diagnosis	Yes (%)	21.5 % (4/19)	Present at diagnosis: 5.32 95 % CI [0.85–33.48]	**0.04808**
	No (%)	78.95 % (15/19)		
Estrogen receptor (ER)	Positive (%)	78.95 % (15/19)	ER positive: 0.0995 % CI [0.01–0.56]	**0.001523**
	Negative (%)	21.5 % (4/19)		
Progesterone receptor (PGR)	Positive (%)	68.42 % (13/19)	PGR positive: 0.2195 % CI [0.05–0.92]	**0.02453**
	Negative (%)	31.58 % (6/19)		
Her2/neu	Positive (%)	21.5 % (4/19)	HER2/new positive: 0.83 95 % CI [0.18–3.76]	0.8096
	Negative (%)	78.95 % (15/19)		
Proliferation index Ki67	Positive (%)	89.47 (17/19)	Ki67 positive: 1.43 95 % CI [0.31–6.69]	0.6451
	Negative (%)	10.53 (2/19)		
Modified proliferation index Ki67	Positive (%)	52.63 (10/19)	Ki67 > 20 % positive: 3.25 95 % CI [1.0–10.5]	**0.03831**
	Negative (%)	47.37.5 (9/19)		
β-catenin dependent WNT score	High > 2 (%)	42.11 (8/19)	WNT-Score high: 0.35 95 % CI [0.11–1.12]	0.06487
	Low ≤ 2 (%)	57.89 (11/19)		
β-catenin independent WNT score	High > 4(%)	68.42 % (13/19)	WNT-Score high: 1.71 95 % CI [0.63–4.63]	0.2849
	Low ≤ 4 (%)	31.58 % (6/19)		

Patient cohort was characterized according to listed parameters in the first column. Type of classification and distribution within the cohort as well as impact on survival including *p* value (logrank) is given for each parameter

Bold and underlined *p*-values are meant to highlight those below 0.05

**Table 2:** Univariate analysis of clinicopathological baseline data and IHC markers derived from breast cancer liver metastases and their effect on overall survival (OS after occurrence of liver metastasis)

Parameter	Classification	Distribution	Impact on survival Hazard ratio [95 % CI]	*p* value (logrank)
Type of surgery	Resection (%)	20.58 % (7/34)	Punch: 3.36 95 % CI [1.14–9.88]	**0.0207**
	Punch (%)	79.41 % (27/34)		
Other distant metastases present at liver metastasis	Yes (%)	44.12 % (15/34)	Present at diagnosis: 1.70 95 % CI [0.80–3.65]	0.1662
	No (%)	55.88 % (19/34)		
Chemotherapy (CTx) after diagnosis of liver metastasis	Yes (%)	86.21 % (25/29*)	Yes: 2.21 95 % CI [0.65–7.50]	0.1911
	No (%)	13.79 % (19/29*)		
Estrogen receptor (ER)	Positive (%)	55.88 % (19/34)	ER positive: 0.87 95 % CI [0.42–1.80]	0.7003
	Negative (%)	44.12 % (15/34)		
Progesterone receptor (PGR)	Positive (%)	32.35 % (11/34)	PR positive: 0.93 95 % CI [0.43–1.99]	0.8428
	Negative (%)	67.65 % (23/34)		
Her2/neu	Positive (%)	26.47 % (9/34)	HER2/neu positiv: 1.06 95 % CI [0.45–2.50]	0.8997
	Negative (%)	73.53 % (25/34)		
Proliferation index Ki67	Positive (%)	91.18 % (31/34)	Ki67 positive: 2.79 95 % CI [0.65–11.92]	0.1481
	Negative (%)	8.82 % (3/34)		
Modified proliferation index Ki67	Positive (%)	52.94 % (18/34)	Ki67 > 30 % positive: 2.46 95 % CI [1.11–5.44]	**0.0222**
	Negative (%)	47.06 % (16/34)		
β-catenin dependent WNT score	High > 1 (%)	44.12 % (15/34)	Wnt-Score high: 0.74 95 % CI [0.35–1.55]	0.4179
	Low ≤ 1 (%)	55.88 % (19/34)		
β-catenin independent WNT score	High > 5(%)	64.71 % (22/34)	Wnt-Score high: 2.19 95 % CI [1.02–4.69]	**0.0391**
	Low ≤ 5 (%)	35.29 % (12/34)		

Patient cohort was characterized according to listed parameters in the first column. Type of classification and distribution within the cohort as well as impact on survival including *p* value (logrank) is given for each parameter

Bold and underlined *p*-values are meant to highlight those below 0.05

* Cases where not for all patients baseline data was available

All patients were diagnosed with breast cancer between 1973 and 2011 at a median age of 57.5 years, 95 % CI [41.9–76.6]. On diagnosis, patients were staged as follows: UICC I: three patients, UICC II; 19 patients, UICC III: six patients, UICC IV: five patients (one patient was missing UICC data). All patients with metachronic hepatic metastases developed these with a time to 50 % at risk of 87.1 months 95 % CI [52.5–109.2]. Individual treatment strategies are summarized in Supplemental Table 2.

The OS had a time to 50 % at risk of 95.5 months 95 % CI [73.6–127.9]. Survival after diagnosis of liver metastasis (OS liver metastasis) revealed a time to 50 % at risk of 15.91 months 95 % CI [9.83–27.06].

The presence of extrahepatic distant metastases on diagnosis lead to a significantly shorter survival (HR 5.32 95 % CI [0.85–33.48], *p* = 0.0481). Patients who underwent surgery for resectable liver metastases (6/34, 17.6 %) had a significantly improved survival compared to patients without liver surgery (HR 3.36 95 % CI [1.14–9.88], *p* = 0.0207) (Supplemental Fig. 1A). Survival was significantly shorter in older patients at the time of diagnosis of breast cancer (median age 57.5; age >57.6: HR 2.16 95 % CI [1.02–4.57], *p* = 0.0405).

### Expression of hormone receptors

ER and PGR expression were detected in 15/19 (78.95 %) and 13/19 (68.42 %) of the breast cancer primaries and in 19/34 (55.88 %) and 11/34 (32.35 %) of the liver metastases. 4/19 (21.05 %) of the primaries demonstrated HER-2 positivity, whereas in the liver metastases 9/34 (26.47 %) were positive. Focusing on the 19 available pairs of matched primaries and liver metastases, the expression of ER was significantly lower in the metastases (primaries 15/19 (78.95 %); metastases: 11/19 (57.89 %) and significantly correlated (r = 0.51, 95 % CI [0.07–0.78], *p* = 0.02). In the matched samples, 13/19 (68.42 %) of the breast cancer primaries revealed PGR expression, whereas only 6/19 (31/0.59 %) liver metastases were positive. Thus, PGR expression also decreased during the development of liver metastasis, however no correlation was detected (r = 0.16, 95 % CI [−0.32–0.57], *p* = 0.52). Within the matched samples, HER-2 expression was 21.05 % in primaries: 4/19 and 26.32 % in metastases: (5/19) and significantly correlated (r = 0.86, 95 % CI [0.67–095], *p* = 1.85 × 10^−6^).

**Fig. 3:**
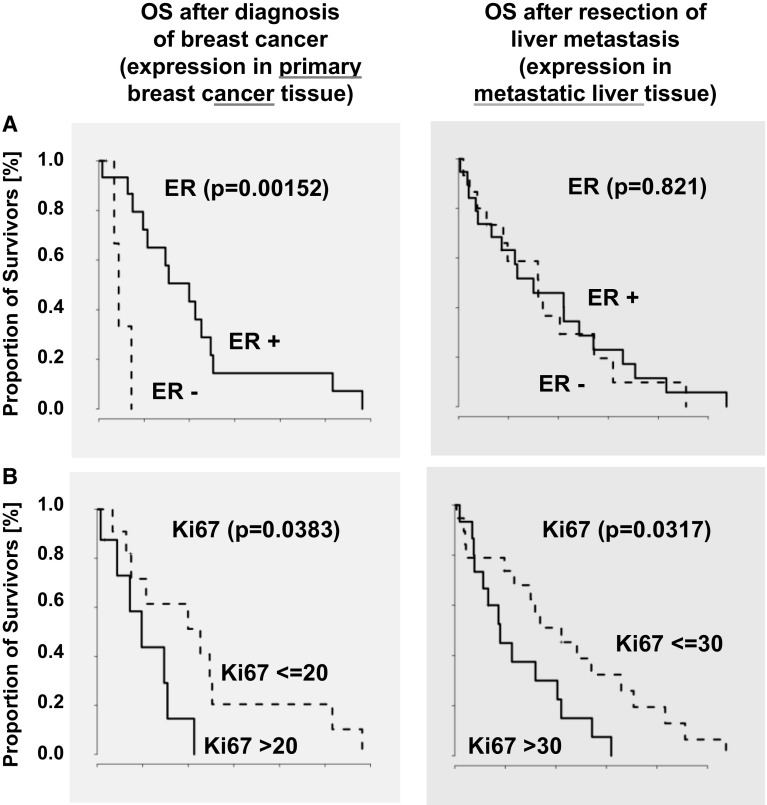
Kaplan-Meier curves illustrate that ER expression in the primary tumor but not in the liver metastases is correlated with a good prognosis (**a**). Positivity for the proliferation index Ki67 in the primary tumor tissue as well as in the liver metastases (**b**) is associated with reduced overall survival

Thus, the expression of estrogen and progesterone receptor in the breast cancer primaries is associated with better survival analyzed from the time point of first diagnosis (ER HR: 0.09 95 % CI [0.01–0.56], *p* =  0.0015 and PGR HR: 0.21 95 % CI [0.05–0.92], *p* = 0.0245) (Fig. 3a and Table 1). In contrast, in the liver metastases the expression of PGR and ER is no longer prognostic analyzed from the time point after liver metastasis resection/biopsy (Fig. 3a; Table 2).

### High proliferation index Ki67 in primary tumors and metastases was associated with shorter survival

In the primaries, the proliferation index Ki67 ranged from 5 to 80. 17/19 (89.47 %) demonstrated a proliferation index of Ki67 ≥ 10 %. In 31/34 (91.18 %) of the liver metastases, a proliferation index of Ki67 [10 %] was detected with a range from 0 to 80. Owing to the fact that almost all primary and metastases samples were positive for the proliferation index Ki67, the median expression was taken as cutoff to perform survival analyses. Taking the primaries’ median proliferation index Ki67 of 20 %, a higher proliferation index Ki67 was associated with shorter survival (HR 3.25 95 % CI [1.0–10.5], *p* = 0.0383). The same is true for the liver metastases (median 30 %), for which survival was significantly shorter when Ki67 staining was above the median (HR 2.46, 95 % CI [1.11–5.44], *p* = 0.0222) (Fig. 3b).

### Expression of WNT markers in primaries and metastases

**Fig. 4:**
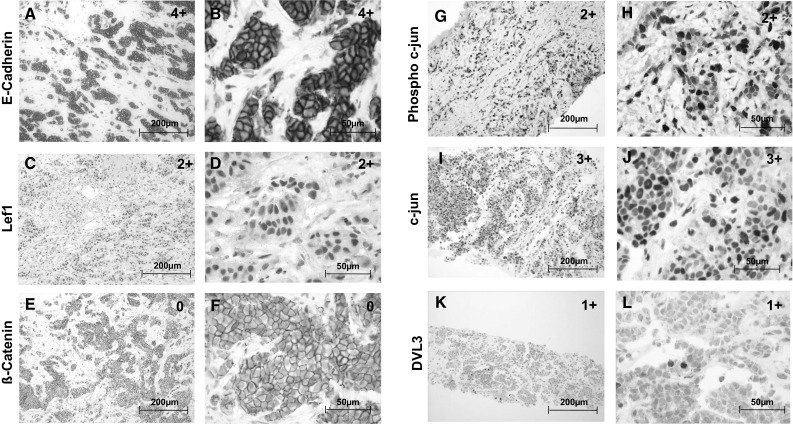
Immunohistochemical stainings for all proteins analysed were performed on primary tumors and metastases respectively. Representative pictures are shown for stainings of primary tumor samples (**a**–**f**) and on metastatic tissue (**g**–**l**). IHC-staining of membrane E-cadherin showing a positivity in primary tumor cells of >75 % in 20× (**a**) and 40× magnification (**b**); nuclear Lef1 positivity is shown at 20× (**c**) and 40× magnification of primary tumor tissue being positive in 30 % of tumor cells (**d**); β-catenin staining is shown in panel **e** (20×) and **f** without any nuclear activity. Representative examples of nuclear Phospho-c-Jun staining is shown in **g** (20×) and **h** (40×) with a positivity rate of >26 % for intrahepatic breast cancer metastases cells; nuclear c-Jun staining is represented in panels **i** (20×) and **j** (40×) showing positive stained nuclei in >76 % of metastatic tissue; nuclear Dvl3 staining is also represented in liver metastases beeing positive in <25 % of metastases cells at a magnification of 20× (**k**) and 40× (**l**). For all markers, the specific expression pattern was analyzed for primaries and metastases and classified into five groups: 0 = 0 %, 1+ = 1–25 %, 2+ = 26–50 %, 3+ = 51–75 % and 4+ = 76–100 % of the tumor cells with positive (≥1) staining. Bar: 200 μm (**a**, **c**, **e**, **g**, **i** and **k**) and 50 μm (**b**, **d**, **f**, **h**, **j** and **l**)

E-cadherin expression was detected in all 19/19 (100 %) of the breast cancer primaries. Three samples were graded as 1+, one sample as 2+ and fifteen samples as 4+ (Fig. 4a + b). In the liver metastases, 30/34 (88.23 %) of the samples stained positive for E-cadherin. Apart from two samples graded as 1+ and 3+ respectively, all other 28 samples were graded as 4+. Nuclear detection of β-catenin was neither possible in the primaries (0 %) nor in the liver metastases (0 %) (Fig. 4e + f). Nuclear c-Jun was detected in 18/19 (94.73 %) of the breast cancer primaries. Three samples were graded as 1+, eight as 2+, five as 3+ and two as 4+. All the liver samples (34/34) expressed nuclear c-Jun. Five samples were graded as 1+, ten as 2+, thirteen as 3+ and six as 4+ (Fig. 4i + j). Nuclear Phospho-c-Jun was expressed in 18/19 (94.73 %) of the primary breast cancer samples. Fifteen samples were graded as 1+, and two samples with 2+ and 3+, respectively.

In the liver metastases, 32/34 (94.11 %) expressed nuclear Phospho-c-Jun. Twenty-one samples were graded as 1+, eight as 2+, and two samples as 3+ and 4+, respectively (Fig. 4g + h).

Nuclear Lef1 expression was detected in 18/19 (94.73 %) of the primary samples (Fig. 4c + d). Four samples were graded as 4+, nine as 2+, three as 3+ and two as 4+. In the liver metastases, nuclear Lef1 expression was detected in 29/34 (85.29 %) of the samples. Sixteen samples were graded as 1+, eight as 2+, three as 3+ and two as 4+. Nuclear Dvl3 was detected in 15/19 (78.94 %) of the primaries. Nine samples were graded as 1+, five as 2+ and one as 3+. In the liver metastases, 33/34 (97.05 %) were positive for nuclear Dvl3. Twenty-four were graded as 1+, six as 2+, two as 3+ and one as 4+ (Fig. 4k + l).

### WNT score was associated with shorter survival

**Fig. 5:**
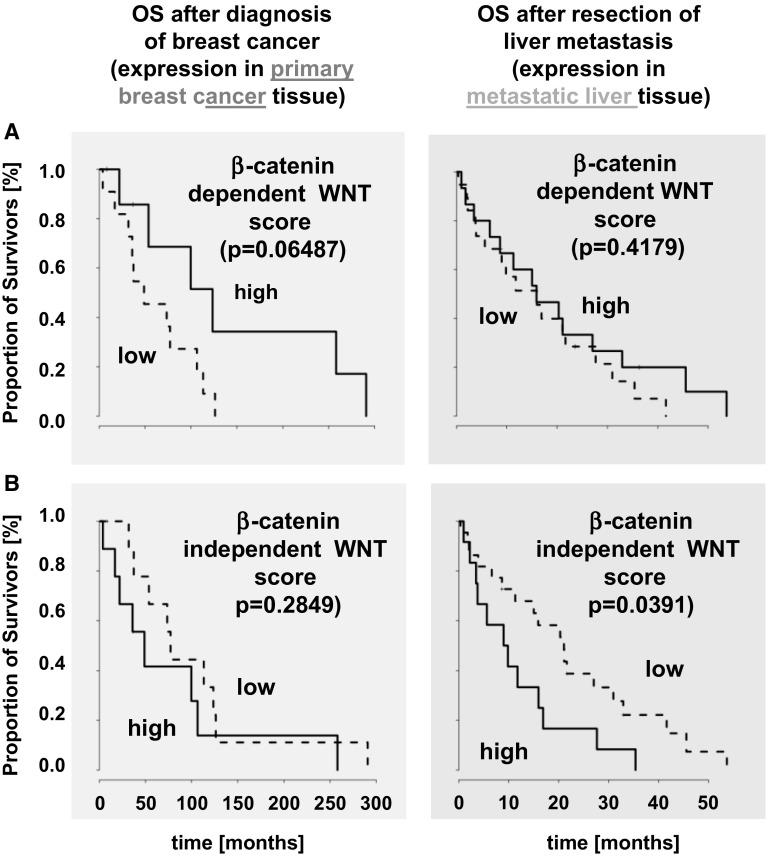
No prognostic impact could be shown for the β-catenin-dependent WNT score (**a**). In contrast, the β-catenin-independent WNT score has prognostic impact on survival in the metastatic setting (**b**). Survival rates are depicted with Kaplan–Meier-curves in months

The median β-catenin-dependent WNT score (representing β-catenin-dependent WNT signaling) was two (95 % CI [0.45–6.1]) in the primary tumors and one (95 % CI [0.0–7.0]) in the liver metastases. The median β-catenin-independent WNT score was four (95 % CI [1.45–8.1]) in the primaries and five (95 % CI [2.7–9.2]) in the liver metastases (Supplemental Fig. 3). In the matched liver metastases samples, the β-catenin-dependent WNT score decreased to one (95 % CI [0.0–7.0]) and the β-catenin-independent WNT score increased to 5 (95 % CI [1.9–9.1]). In the breast cancer primaries, neither the β-catenin-dependent WNT score (HR 0.35 95 % CI [0.11–1.12], *p* = 0.0649) nor the β-catenin-independent WNT score alone (HR 1.71 95 % CI [0.63–4.63], *p* = 0.2849) were of prognostic value. In contrast, in the liver metastases, a high β-catenin-independent WNT score (HR 2.19 95 % CI [1.02–4.69], *p* = 0.0391) proved to be unfavorable analyzed from the time point after liver metastasis, whereas the β-catenin-dependent WNT score was not prognostic (HR 0.74 95 % CI [0.35–1.55], *p* = 0.4179) (Fig. 5a + b). Taken together, the IHC results revealed a significance of β-catenin-independent WNT signaling during liver metastasis which is correlated with an unfavorable prognosis at the time point of resection/biopsy of the liver metastasis. In contrast, a high β-catenin-dependent WNT score has a tendency for better OS in the primary, while at the time point of liver metastasis the score is not correlated with prognosis any more.

## Discussion

The liver is the second most frequent site of metastasis in breast cancer patients. Furthermore, liver metastases remain associated with an unfavorable prognosis and there is an urgent need to improve the therapeutic options. Along this line, both this and previous studies indicate the potential benefit of resecting solitary breast cancer liver metastases as already established for patients with metastatic colorectal cancer [3, 47–49]. However, all reports available are retrospective and included small patient cohorts; randomized or even prospective trials are still missing. In a multimodal tailored approach, it is crucial to identify patient subgroups that benefit from specific treatment options including surgical resection, based on distinct biomarker profiles or prognostic parameters.

In the current study, we investigated both well-established and innovative biomarkers in synchronous (n = 5) or metachronous (n = 29) liver metastases of breast cancer patients as well as in available matched primary tumors (n = 19). While biomarker analysis identified the proliferation index Ki67 to be prognostic in both types of tissue (primary and metastatic), ER status was shown to lose its prognostic value: Surprisingly, ER positivity in the metastatic samples was not prognostic anymore analyzed from the time point of liver metastasis, although it was prognostic in the matched primary tumors analyzed from the time point of the first diagnosis of the primary as expected. This discrepancy may be attributed to the fact that metastatic breast cancer cells are more resistant to anti-hormonal treatment than non-metastatic cells [50]. Furthermore, the unique microenvironment of the metastatic liver tissue could be a second decisive parameter [51]. Previously, significant differences in treatment response in a mouse model could be observed, dependent on the anatomical injection sites. While various subcutaneously injected cancer cells responded very well to immune therapy, the response was less pronounced in the orthotopic models [52]. However, no studies have been undertaken comparing the treatment response of estrogen-receptor-positive breast cancer cells injected at various anatomical sites (breast versus liver or lung) and treated subsequently with Tamoxifen or other anti-hormonal drugs.

Additionally, current investigations detected a genomic evolution with gain of, tissue-specific and de novo mutations in the metastatic samples in comparison to the primary tumor [53–56]. This subsequently leads to phenotypic changes of the malignant cells as well as changes in the activity of directly and indirectly affected pathways. Recently, a significant overexpression of HER-3 was described for brain metastasis. Interestingly, the ligand NRG1/2 was barely expressed in the metastatic cells, thus the ligand seems to derive from the brain microenvironment [57]. This again indicates a very important influence of the microenvironment of the affected organ.

Nonetheless, both (genetic) adaptation of the tumor cells to the metastatic host organ during the process of metastasis and the influence of the new microenvironment are very obvious explanations for our finding that ER expression as a biomarker changes its prognostic capacity in the metastatic tissue.

In this line, the β-catenin-independent WNT score gains prognostic impact in the metastatic tissue of the liver, which further implies the above described mechanisms, in particular the adaptation during the process of metastasis and to the new host microenvironment. Both processes presuppose an enormous plasticity of the metastatic cells with subsequent differential gene/protein expression at the different metastatic steps and sites of metastases. While during progression in the primary tumor, pre-metastatic cells have to gain mesenchymal characteristics (EMT), they have to regain epithelial features (MET) and polarization of the pre-metastatic cells in the newly nascent metastatic tissue at the distant sites. The latter process is well described during embryonic development for the establishment of the mesoderm. In that case, mesenchymal cells also regain epithelial characteristics (MET) in a mesenchymal neighborhood that is WNT/PCP-signaling-dependent. Furthermore, WNT/PCP-signaling regulates not only the establishment but also the maintenance of the epithelial polarity and orientation of a single cell in the overall context of the tissue. Recent findings of β-catenin-independent WNT components in cancer also strengthen their role during metastasis. For example, in breast cancer, the WNT/PCP pathway was essential in the tumor-stroma communication via exosomes to gain metastatic features [58]. We demonstrated that macrophage- and microglia-derived WNT-signaling enhanced breast cancer invasion in a β-catenin-independent way [18, 21, 59]. The latter interaction additionally supported the colonization of the brain tissue [18, 21] and we described overexpression of c-Jun and ROR1/ROR2 in metastatic brain tissue of breast cancer patients while detecting no nuclear β-catenin [23]. This is in line with our in vitro findings that, while total and active β-catenin were detectable in the benign MCF-7 cell line and the invasive, basal-like breast cancer cell line MDA-MB-231, the expression of β-catenin-independent WNT ligands seemed to increase invasive- and aggressiveness (MCF-7 < SK-BR3 < MDA-MB-231). All these findings underline the role of β-catenin-independent signaling in the later stages of metastasis. Moreover, there is an increase of the WNT-β-catenin-independent score from the primary to the liver metastasis. Current genomic investigations also detected an evolution with gain of tissue-specific and de novo genetic mutations in the metastatic samples in comparison to the primary tumor [53–55, 60, 61]. This subsequently leads to phenotypic changes of the malignant cells as well as changes in the activity of directly and indirectly affected pathways. Recently, it was furthermore described for brain metastasis a significant activation of HER-3. Interestingly, the ligand NRG1/2 was barely expressed in the metastatic cells, thus the ligand seems to derive from the brain microenvironment [57].

All these findings underline the role of β-catenin-independent signaling in the later stages of metastasis. However, so far this has received little attention compared to WNT/β-catenin signaling.

However, further studies are required for a more specific characterization of the role of the microenvironment in the development of liver metastases and liver-specific mutations or pathway activations. Here we clearly demonstrate a role of the WNT/β-catenin-independent signaling pathway during this process.

Taken together, the presented data clearly demonstrate that biomarkers derived from studies in primary tumors cannot simply be translated to the metastatic tissue because of the parallel evolution of the metastatic cells and the organ-specific growth conditions accountable by their specific microenvironments. Furthermore, we revealed the significance of β-catenin-independent WNT signaling at least for liver metastasis. However, so far the WNT/β-catenin independent signaling has received little attention compared to WNT/β-catenin signaling. In conclusion, we suggest that prognostic biomarkers should be implemented in the clinical decision making or stratification of patients with liver metastasis of breast cancer in multimodal treatment strategies. This may include the resection of breast cancer liver metastases in selected patients.

## Electronic supplementary material

Supplementary material 1 (DOCX 852 kb) Supplemental Fig. 1: **(A)** Kaplan–Meier curves depicting cancer-specific survival in month for all patients. Patients with limited metastatic spread being resected had a better long-term survival (*p* = 0.0207) compared to non-resected patients. **(B)** Workflow of this study showing the included patient cohort and tissues available of breast cancer primaries and liver metastases

Supplementary material 2 (DOCX 823 kb) Supplemental Fig. 2: **(A)** Immunoblot of active β-catenin expression in cytosolic (C) and nuclear (N) fractions of MCF-7, SK-BR-3 and MDA-MB-231 cells. HDAC is included as control for successful fractionation. **(B)** Immunofluorescence staining of ROR2 (green) in MCF-7 empty vector (ctl) and ROR2-overexpressing (pROR2) cells. F-Actin (Phalloidin) staining (red) was used to visualize the cytoskeleton of the cells. **(C)** Measurement of the cell index as a marker for cell proliferation via xCELLigence for MCF-7 and SK-BR-3 empty vector (ctl) and pROR2 cells. One representative image out of three biologically and technically independent experiments is shown. **(D)** Expression of active β-catenin, total β-catenin and HDAC in cytosolic (C) and nuclear (N) fractions of MCF-7 empty vector and ROR2-overexpressing cells was analyzed by immunoblotting. **(E)** Flow cytometry of MDA-MB-231 cells (gray = isotype control, green = stained cells with ROR1 knockdown, shROR1, black = stained cells nonsense control, ns ctl) confirming the knockdown of ROR1. (F) Immunoblot analysis of ROR2 expression in MDA-MB-231 nonsense control (ctl) and ROR1 knockdown (shROR1) cells. Pos. Ctl = positive control. (G) MCF-7 cells were transiently transfected with ROR2-Flag and/or myc-tagged Dvl1, Dvl2 or Dvl3 and stimulated with either control supernatant (sn) or Wnt5a-conditioned medium, which was collected from 3T3murine fibroblasts transduced with pMSCV-Xenopus WNT-5a or an empty control. Lysates were co-immunoprecipitated using anti-Flag magnetic beads and precipitated proteins were detected with anti-myc and anti-Flag antibodies. The bottom lanes show the input levels in the lysates

Supplementary material 3 (DOCX 22 kb) Supplemental Fig. 3: Boxplots depicting the expression of the β-Catenin-dependent WNT score (white box) and the β-catenin-independent WNT score (grey box) score in the primary tumors (left side) and well as in the matched liver metastases (right side)

Supplementary material 4 (DOCX 16 kb)

Supplementary material 5 (DOCX 24 kb)

## Abbreviations

ER: Estrogen receptor
OS: Overall survival
NSCLC: Non-small cell lung cancer
EMT: Epithelial mesenchymal transition
LEF/TCF: Lymphoid enhancer factor/T cell factor
MET: Mesenchymal epithelial transition
RTK: Receptor tyrosine kinases
JNK: Jun-N-terminal kinase
Fz: Frizzled
DVL: Dishevelled
WNT/PCP: WNT/planar cell polarity pathway
PGR: Progesterone receptor
HER-2: Erb-b2 receptor tyrosine kinase
FFPE: Formalin-fixed paraffin-embedded
IHC: Immunohistochemistry
UICC: Union Internation Contre le Cancer
C/S-ISH: Chromogene or silver in situ hybridization
HER-3: Erb-b3 receptor tyrosine kinase
NRG1/2: Neuregulin 1/2
ROR1/2: Receptor tyrosine kinase-like orphan receptor
