# Supplementary material for: β-catenin-independent WNT signaling and Ki67 in contrast to the estrogen receptor status are prognostic and associated with poor prognosis in breast cancer liver metastases
Source: Clin Exp Metastasis. 2016 Feb 9;33:309–23. doi: 10.1007/s10585-016-9780-3 (PMC4799797; doi:10.1007/s10585-016-9780-3)
Supplement: Supplementary file 4 — Supplementary material 4 (DOCX 16 kb) [file 10585_2016_9780_MOESM4_ESM.docx]

Supplemental Table 1: Primer Sequences

| **gene** | **forward (5’ – 3’)** | **reverse (5’ – 3’)** |
| --- | --- | --- |
| CTNNB1 | ATCTGTCTGCTCTAGTAATAAGCC | CTGAAAGATTCCTGAGAGTCCA |
| GNB2L1 | AACCCTATCATCGTCTCCT | CAATGTGGTTGGTCTTCAG |
| HPRT1 | TATGCTGAGGATTTGGAAAGG | CATCTCCTTCATCACATCTCG |
| ROR1 | CGTCTATATGGAGTCTTTGCAC | GAATGGCGAACTGAGAACAC |
| ROR2 | TTCTTCTTGGTTTGCATGTG | CTGATCTCTTTGAGTTTGGC |
