# Supplementary material for: β-catenin-independent WNT signaling and Ki67 in contrast to the estrogen receptor status are prognostic and associated with poor prognosis in breast cancer liver metastases
Source: Clin Exp Metastasis. 2016 Feb 9;33:309–23. doi: 10.1007/s10585-016-9780-3 (PMC4799797; doi:10.1007/s10585-016-9780-3)
Supplement: Supplementary file 5 — Supplementary material 5 (DOCX 24 kb) [file 10585_2016_9780_MOESM5_ESM.docx]

| **Patient** | **Age at diagnosis** | **Histology** | **Surgery Date** | **TNM-Stage** | **UICC** | **Recurrence Date** | **Metastases** | **Liver Metastases Date** | **Biopsy/ Surgery Liver** | **Systemic treatment of Metastases** |
| --- | --- | --- | --- | --- | --- | --- | --- | --- | --- | --- |
| **1** | **60** | **ductal** | **15.03.2000** | **pT1cpN1M0** | **IIA** | **01.06.2002** | **HEP+ LYM** | **01.06.2002** | **B** | **CTx** |
| **2** | **50** | **ductal** | **15.02.2000** | **pT2pN1M0** | **IIB** | **15.09.2002** | **HEP** | **15.09.2002** | **B** | **CTx** |
| **3** | **57** | **ductal, lobular** | **22.01.2002** | **pT2pN1M0** | **IIB** | **15.03.2005** | **HEP+ Breast** | **15.03.2005** | **B+(S)** | **CTx** |
| **4** | **72** | **ductal** | **24.08.2005** | **TxNxM1** | **IV** | **22.08.2005** | **HEP** | **22.08.2005** | **B** | **NA** |
| **5** | **59** | **NA** | **15.01.1989** | **pT3pN0M0** | **IIB** | **23.01.2006** | **HEP** | **23.01.2006** | **B** | **CTx** |
| **6** | **45** | **ductal, lobular** | **15.11.2001** | **pT1cpN0M0** | **IA** | **15.01.2003** | **OSS** | **30.03.2006** | **B** | **CTx** |
| **7** | **46** | **lobular** | **02.09.2002** | **pT3pN1pMx** | **IIIA** | **05.03.2007** | **HEP** | **05.03.2007** | **S** | **None** |
| **8** | **57** | **ductal** | **08.10.1999** | **pT1cpN0M0** | **IA** | **15.04.2007** | **HEP** | **15.04.2007** | **B** | **CTx** |
| **9** | **46** | **ductal, lobular** | **29.08.2002** | **pT2pN1M0** | **IIB** | **15.04.2007** | **HEP** | **15.04.2007** | **B** | **CTx** |
| **10** | **64** | **ductal** | **10.10.2005** | **pT1cpN1M0** | **IIA** | **08.06.2007** | **HEP** | **08.06.2007** | **B** | **CTx** |
| **11** | **42** | **ductal** | **09.06.1998** | **pT2pN1M0** | **IIB** | **13.07.2007** | **HEP** | **13.07.2007** | **B** | **CTx** |
| **12** | **64** | **ductal, lobular** | **09.06.2009** | **pT2pN2M1** | **IV** | **29.05.2009** | **HEP** | **29.05.2009** | **B** | **NA** |
| **13** | **62** | **ductal** | **29.05.2008** | **pT1cpN1M0** | **IIA** | **15.06.2009** | **HEP** | **15.06.2009** | **B** | **CTx** |
| **14** | **45** | **ductal** | **24.02.1986** | **pT2pN0M0** | **IIA** | **01.05.2007** | **Breast+OSS** | **15.08.2009** | **B** | **CTx** |
| **15** | **58** | **ductal** | **24.08.2010** | **cT2cN0M1** | **IV** | **15.02.2010** | **HEP** | **15.02.2010** | **B** | **None** |
| **16** | **64** | **ductal, lobular** | **21.03.2007** | **pT2pN0M0** | **IIA** | **15.04.2010** | **HEP+OSS** | **15.04.2010** | **B** | **None** |
| **17** | **73** | **ductal** | **10.09.2002** | **pT2pN0M0** | **IIA** | **28.04.2010** | **HEP+OSS** | **28.04.2010** | **B** | **CTx** |
| **18** | **53** | **ductal, lobular** | **03.08.2001** | **pT2pN1M0** | **IIB** | **15.08.2010** | **HEP+OSS** | **15.08.2010** | **B** | **CTx** |
| **19** | **75** | **ductal, inflammatory** | **24.10.2011** | **pT4pN2M1** | **IV** | **21.06.2011** | **HEP** | **21.06.2011** | **S** | **NA** |
| **20** | **51** | **ductal** | **15.01.1996** | **pT2pN1M0** | **IIB** | **15.01.2004** | **HEP** | **15.01.2004** | **B+S** | **CTx** |
| **21** | **49** | **NA** | **15.11.1997** | **pT1cpN0M0** | **IA** | **15.02.1998** | **HEP** | **15.02.1998** | **B** | **CTx** |
| **22** | **68** | **ductal** | **15.11.2005** | **TxN3M0** | **IIIC** | **01.03.2006** | **HEP** | **01.03.2006** | **S** | **CTx** |
| **23** | **44** | **NA** | **15.04.1996** | **pT1cpN1M0** | **IIA** | **15.08.2002** | **PER** | **03.03.2005** | **B** | **CTx** |
| **24** | **67** | **ductal** | **07.04.1996** | **pT4pN2M0** | **IIIB** | **01.12.2006** | **HEP+OSS** | **01.12.2006** | **B** | **CTx** |
| **25** | **67** | **ductal** | **15.03.1992** | **pT1cpN1pM0** | **IIA** | **15.07.1995** | **PLE** | **15.03.2007** | **B** | **CTx** |
| **26** | **56** | **lobular** | **15.07.2000** | **pT4bpN1M0** | **IIIB** | **29.01.2004** | **OSS** | **15.10.2007** | **B** | **NA** |
| **27** | **48** | **ductal** | **15.05.1995** | **pT3pN1M0** | **IIIA** | **15.10.2006** | **HEP** | **15.10.2006** | **B+S** | **CTx** |
| **28** | **79** | **ductal** | **26.02.2009** | **TxNxM1** | **IV** | **09.03.2009** | **HEP** | **09.03.2009** | **B** | **None** |
| **29** | **53** | **lobular** | **15.11.2002** | **pT2pN1M0** | **IIB** | **23.03.2009** | **HEP** | **23.03.2009** | **B** | **CTx** |
| **30** | **37** | **ductal** | **15.07.1973** | **TxNxMx** | **NA** | **16.06.2009** | **HEP+PUL+OSS+LYM** | **16.06.2009** | **B** | **CTx** |
| **31** | **61** | **ductal** | **15.07.1992** | **pT2pN0M0** | **IIA** | **01.12.2008** | **Breast** | **15.11.2009** | **B** | **CTx** |
| **32** | **49** | **ductal** | **14.11.2007** | **pT1cpN2M0** | **IIIA** | **15.01.2009** | **HEP** | **15.01.2009** | **B** | **CTx** |
| **33** | **51** | **ductal** | **19.03.2009** | **pT2pN0M0** | **IIA** | **02.07.2010** | **HEP+Breast** | **02.07.2010** | **B+S** | **CTx** |
| **34** | **47** | **NA** | **15.10.1992** | **pT2pN0M0** | **IIA** | **15.07.2001** | **OSS** | **15.07.2006** | **B** | **CTx** |

**Supplemental Table 2: Patient cohort**

Supplement Table 1 shows the patient cohort with clinical data and treatment details. (Legend: CTx-Chemotherapy; HEP–Liver; LYM-Lymphnode; OSS–Bone; PER–Peritoneum; PUL–Lung; B-punch biopsy; S-Surgery; NA-not available.
