# Supplementary material for: β-catenin-independent WNT signaling and Ki67 in contrast to the estrogen receptor status are prognostic and associated with poor prognosis in breast cancer liver metastases
Source: Clin Exp Metastasis. 2016 Feb 9;33:309–23. doi: 10.1007/s10585-016-9780-3 (PMC4799797; doi:10.1007/s10585-016-9780-3)
Supplement: Supplementary file 3 [file 10585_2016_9780_MOESM1_ESM.docx]

Supplemental Figure 1

Proportion of Survivors [%]

0.6

0.8

1.0

0.4

0.2

0.0

10

time [months]

0

20

30

40

50

A

No Resection

Resection

B

p=0.0207

Matched primary tumors (n=19) 

Synchronous (n=5) and metachronous (n=29) liver metastases

Breast cancer patients with liver metastases and clinical long-term Follow-up (N=34) 

Resection specimen (n=19)

Characterization of hormone receptor and HER-2 status and staining for WNT signaling components. Subsequent correlation with long-term outcome. 

Punch biopsy 

(n=27)

Resection specimen (n=7)

ctl

B

pROR2

C

Phalloidin

ROR2

Phalloidin

ROR2

Supplemental Figure 2

E

ROR1 PE

cell counts

Isotype ctl

ns ctl

shROR1

ROR2

120kDa

Ponceau

MDA-MB-231

ctl

shROR1

Tubulin

pos.

ctl

52kDa

F

act. β-cat

92kDa

Ponceau

HDAC

60kDa

MCF-7

SK-BR-3

MDA-MB-231

A

D

ctl

pROR2

HDAC

Ponceau

β-catenin

act. β-cat

92kDa

92kDa

60kDa

ctl

pROR2

MCF-7

SK-BR-3

MCF-7

SK-BR-3

C

G

myc

Flag

myc

Flag

75kDa

75kDa

50kDa

50kDa

lysate

IP anti-Flag

ROR2-Flag

Myc-Dvl1

Myc-Dvl2

Myc-Dvl3

control sn

Wnt5a sn

+

+

+

+

+

+

+

+

+

+

+

+

+

+

+

+

+

+

+

+

+

+

C

C

C

N

N

N

C

N

C

N

C

N

C

N

cell counts

cell counts

Time [h]

Time [h]

Supplemental Figure 3

6

8

10

4

2

ß-catenin

dep.

WNT score 

Primary

Metastases

WNT Scores

ß-catenin

indep.

WNT score  

ß-catenin

dep.

WNT score 

 

ß-catenin

indep. WNT score  

0

12
