# Supplementary material for: β-catenin-independent WNT signaling and Ki67 in contrast to the estrogen receptor status are prognostic and associated with poor prognosis in breast cancer liver metastases
Source: Clin Exp Metastasis. 2016 Feb 9;33:309–23. doi: 10.1007/s10585-016-9780-3 (PMC4799797; doi:10.1007/s10585-016-9780-3)
Supplement: Supplementary file 4 [file 10585_2016_9780_MOESM2_ESM.docx]

ctl

B

pROR2

C

Phalloidin

ROR2

Phalloidin

ROR2

Supplemental Figure 2

E

ROR1 PE

cell counts

Isotype ctl

ns ctl

shROR1

ROR2

120kDa

Ponceau

MDA-MB-231

ctl

shROR1

Tubulin

pos.

ctl

52kDa

F

act. β-cat

92kDa

Ponceau

HDAC

60kDa

MCF-7

SK-BR-3

MDA-MB-231

A

D

ctl

pROR2

HDAC

Ponceau

β-catenin

act. β-cat

92kDa

92kDa

60kDa

ctl

pROR2

MCF-7

SK-BR-3

MCF-7

SK-BR-3

C

G

myc

Flag

myc

Flag

75kDa

75kDa

50kDa

50kDa

lysate

IP anti-Flag

ROR2-Flag

Myc-Dvl1

Myc-Dvl2

Myc-Dvl3

control sn

Wnt5a sn

+

+

+

+

+

+

+

+

+

+

+

+

+

+

+

+

+

+

+

+

+

+

C

C

C

N

N

N

C

N

C

N

C

N

C

N

cell counts

cell counts

Time [h]

Time [h]
