# Supplementary material for: β-catenin-independent WNT signaling and Ki67 in contrast to the estrogen receptor status are prognostic and associated with poor prognosis in breast cancer liver metastases
Source: Clin Exp Metastasis. 2016 Feb 9;33:309–23. doi: 10.1007/s10585-016-9780-3 (PMC4799797; doi:10.1007/s10585-016-9780-3)
Supplement: Supplementary file 5 [file 10585_2016_9780_MOESM3_ESM.docx]

Supplemental Figure 3

6

8

10

4

2

ß-catenin

dep.

WNT score 

Primary

Metastases

WNT Scores

ß-catenin

indep.

WNT score  

ß-catenin

dep.

WNT score 

 

ß-catenin

indep. WNT score  

0

12
